# Guest editorial: Medical bionics: from emerging technologies to clinical practice

**DOI:** 10.1049/htl.2020.0047

**Published:** 2020-06-17

**Authors:** Robert K. Shepherd

**Affiliations:** AM, FAHMS, PhD, Professorial Fellow, Department of Medical Bionics, University of Melbourne; Senior Research Advisor, Bionics Institute

Medical Bionics or neuroprostheses are active implantable devices designed to: (i) provide therapeutic intervention, sensory feedback or motor function via electrical stimulation of nerves or muscles following trauma or disease; and/or (ii) record the electrical activity from nerve or muscle to detect disease states, enable the voluntary control of devices such as prosthetic limbs or provide closed-loop feedback to modulate neural prostheses.

There have been many bionic devices approved for clinical use; the modern neurotechnology industry is dominated by five major commercial applications including: (i) auditory prostheses that provide auditory cues for severe and profoundly deaf; (ii) spinal cord stimulation (SCS) for treatment of chronic back pain; (iii) vagal nerve stimulation for the control of epilepsy and treatment of depression; (iv) deep brain stimulation (DBS) for motor control in Parkinson's disease (PD) and essential tremor, and (v) sacral root stimulation for improved bladder and bowel function. These devices have had a dramatic impact on the quality of life of millions of people around the world and have generated an industry worth US$12.0 billion by 2020 [1].

New neuroprostheses are being developed at an astonishing rate, with successful technologies nurtured under a multidisciplinary environment in order to deliver both safe and effective devices. These technologies are, by necessity, built on advances in engineering, electronics, materials science, electrochemistry, battery technology, neuroscience, clinical and surgical practice, and improved rehabilitation techniques.

The aim of this Special Issue is to bring together eight examples of technological development that hold promise in future clinical application.

**Leccardi and Ghezzi** review the potential use of organic materials in neuroprostheses with specific emphasis on their use at the electrode/neural interface. These polymers offer both mechanical and electrochemical advantages over the use of metal electrodes and can be relatively simply introduced into existing manufacturing techniques. **Richardson and colleagues** review the advantages and limitations of optical stimulation of neural tissue. Direct optical stimulation via infrared light or through the use of optogenetics, have the potential to achieve higher precision of neural activation compared with electrical stimulation. **Kosta, Loizos and Lazzi** present a computational investigation of improved stimulus waveforms for application in retinal prostheses. They describe a class of asymmetric biphasic pulses that can generate precise ganglion cell firing patterns with up to 55% lower current requirements compared to traditional current pulses. **McDermott and Sinclair** review the application and benefit of adaptive DBS in PD by automatically adjusting each patient's stimulation settings in response to their ever-changing needs. A biomarker that estimates the severity of motor impairment and is linked to an adaptive control algorithm to optimises the stimulation is presented. **Parker, Karantonis and Single** describe the closed-loop control of SCSs used to maintain a predetermined level of neural recruitment, and propose a hypothesis to explain the difference in efficacy between open-and closed-loop operational modes. This work provides a rational basis for directing clinical research and improving SCSs. **Kilgore and colleagues** review the optimal architecture of multi-function neuroprostheses for spinal cord injury with particular emphasis on powering strategies. They describe the significant advantages associated with a wired multipoint implant technology with a centralized power supply. **Steadman and Grill** describe novel SCS techniques to restore bladder control in spinal cord injury. While SCS provides a clear pathway to sustainable commercial availability and clinical impact, further research, directed at effective stimulation parameters and the appropriate timing and location of stimulation, is required. Finally, **Parker and Dietz** report on the potential application of SCS to reduce spasticity associated with cerebral palsy. Reviewing the small number of clinical reports using this approach, the authors propose a mechanism of action based on current understanding of SCS and provide techniques that enable improved tuning of these devices for more substantial relief from symptoms.

In closing I would like to thank Professor Christopher James (Editor-in-Chief), Dr Helen Dyball (Executive Editor), and all contributing authors for their generous support of this Special Issue.
